# Does Prior Night’s Sleep Impact Next Day’s Executive Functioning? It Depends on an Individual’s Average Sleep Quality

**DOI:** 10.17505/jpor.2022.24218

**Published:** 2022-06-09

**Authors:** Dian Yu, Carolina Goncalves, Pei-Jung Yang, G. John Geldhof, Laura Michaelson, Yue Ni, Richard M. Lerner

**Affiliations:** 1 Tufts University; 2 National Chengchi University, Taiwan; 3 Oregon State University; 4 American Institutes for Research

**Keywords:** within-person variability, executive functioning, sleep, intensive longitudinal research

## Abstract

Executive functioning (EF) is a series of fundamental goal-directed cognitive abilities that enable effective learning. Differences in daily sleep quality may covary with fluctuations in EF among youth. Most studies linking sleep to EF rely on between-person differences and average effects for the sample. This study employed an intensive longitudinal design and examined the within-person relations between self-reported prior night’s sleep quality and next day’s EF. Students from Grades 4 to 12 (*M* age= 14.60, *SD* = 2.53) completed three behavioral EF tasks repeatedly across approximately one semester. The final analytic sample included 2898 observations embedded in 73 participants. Although, on average, sleep did not significantly covary with EF, there was heterogeneity in within-person sleep-EF relations. Moreover, individuals’ average sleep quality moderated within-person effects. For individuals with low mean sleep quality, a better-than-usual sleep quality was linked to better EF performance. However, for individuals with high mean sleep quality, better-than-usual sleep quality was linked to worse EF performance. Understanding person-specific relations between sleep and EF can help educators optimize EF and learning on a daily basis and produce positive academic outcomes across longer time periods.

Executive functioning (EF) describes a series of complex cognitive functions that support goal-directed behaviors (Blair & Ursache, 2011; Miyake & Friedman, 2012; Miyake et al., 2000). Primary components of EF include working memory, the ability to hold and manipulate information in mind; response inhibition, the ability to withhold one behavior to engage in a different behavior; and cognitive flexibility, the ability to shift attention according to external needs (Dimond, 2006; Garon et al., 2008, Miyake et al., 2000). EF is a fundamental cognitive ability that enables self-regulatory processes and effective learning (Blair & Raver, 2012; Blair & Ursache, 2011; Lantrip et al., 2016; Zelazo & Cunningham, 2007; McClelland et al., 2014; Mills et al., 2018). Studies have linked better EF to better academic achievement, such as math and literary performance (McClelland et al., 2014; Schmitt et al., 2017). Such findings are not surprising because students need adequate EF to pay attention, control their behaviors, and follow teachers’ directions in the classroom to engage in learning activities (Bierman et al., 2008; Raver et al., 2011).

The evidence base regarding the association between EF and learning outcomes has relied primarily on between-person approaches, suggesting that students with higher EF scores are more likely to have better academic achievement. However, learning does not happen during the time of the administration of a specific test. The learning process unfolds across a micro timescale involving minute-to-minute or day-to-day variation. Thus, to optimize learning on a daily basis, it is important to understand how EF change from day to day together with other factors in the developmental context. Sleep quality is a person-specific indicator of an individual’s well-being that might covary with daily EF (e.g., Könen et al., 2015). For instance, understanding how daily EF fluctuations covary with changes in sleep quality might inform micro-time interventions and practices that can optimize daily learning and lead to desirable long-term outcomes. The current study aims at assessing EF repeatedly within individuals and examining the within-person relations between fluctuations in a specific person’s EF and prior night’s sleep quality.

## Within-Person Fluctuations in Executive Functioning

Relational developmental systems (RDS) metatheory suggests that human development unfolds through dynamic and mutually influential relations between individuals and their multi-layered ecology (Bronfenbrenner & Morris, 2006; Overton, 2015). These coactions between individual and ecology (i.e., individual-context coactions) involve biological, cognitive, emotional, and behavioral attributes of the person and their social contexts – all changing interdependently across time (Molenaar, 2014; Overton, 2015; Witherington, 2015). Specific features of the time and place of these coactions for each person create fundamental, person-specific facets of each student’s learning trajectory (Cantor et al., 2021; Lerner, 2018; Rose, 2016). Therefore, a students’ EF capacity might vary across time and place due to the combination of the variation of their personal and contextual factors (Bornstein 2019). Accordingly, using a static score to represent a student’s EF and treating EF as a stable construct may fail to capture the variability and range of a child’s EF capacity.

Changes in EFs can be conceptually differentiated as developmental change or as fluctuations (Ram & Grimm, 2015). Developmental change is usually irreversible and can be seen as transformation (i.e., qualitative change). Such change may be manifested at macro timescales (e.g., months, years). In turn, fluctuations occur at micro timescales (e.g., hours, days) and are often temporary and reversible. Studies have documented fluctuations in EF performance, which is impacted by contextual and personal factors. Some labbased experimental studies of EFs suggest that lab-induced affect, such as anxiety or pleasant mood, can lead to changes in EF performance, suggesting that such variation reflects fluctuations in EFs during a short period of time (Katzir et al., 2010; Lindström & Bohlin, 2012; Oaksford et al., 1996; Phillips et al., 2002). Studies using repeated assessments have also documented meaningful within-person fluctuations in EF in naturalistic settings (Brose et al., 2010, 2012, 2015; Gamaldo et al., 2010; Könen et al., 2015; Kramer et al., 2020; Neubauer et al., 2019; Schmiedek et al., 2009; Yu et al., 2020, 2021). Identifying factors in the context or the person that coact with fluctuations in EF can provide information for optimization EF on a daily basis. Sleep is one personal well-being factor that may coact with EF within individuals.

## Person-Specificity in Relations between Executive Functioning and Sleep

Many studies have demonstrated that differences in sleep are one source of differences in EF development in middle childhood and adolescence (e.g., Andrews et al., 2020; Berger et al., 2019). However, most of the findings are derived from cross-sectional or sparsely repeated (e.g., annual) longitudinal data and statistical analysis of between-person differences. Analyses of such data are typically accompanied by a conclusion that if a child sleeps longer/better than other children in general, they tend to demonstrate better EF than other children (Gradisar et al., 2008; Ortega et al., 2010; Paruthi et al., 2016). Indeed, using data focused on average relations among variables, adequate sleep has been associated with better EF and related outcomes, such as socio-emotional development, as well as positive health behaviors and academic achievement during childhood and adolescence (Araújo & Almondes, 2014; Chen et al., 2006; Galvan, 2019; Gradisar et al., 2008; Kuula et al., 2015; Hayes & Bainton, 2020; Mak et al., 2012; Ortega et al., 2010; Segura-Jiménez et al., 2015; Warren et al., 2016). In turn, insufficient sleep is negatively associated with children and adolescents’ EF skills as well as related abilities, such as engagement in creative and adaptive learning, long-term memory retrieval, problem-solving, and socio-emotional development (Bernier et al., 2014; Nathanson & Beyens, 2018; Sharman & Illingworth, 2020; Short & Chee, 2019; Taveras et al., 2017; Vaughn et al., 2015).

Although most of this literature suggests that sleep is important for EF, not all group-based and average-focused studies of sleep and EF support this conclusion (for reviews, see Chaput et al., 2016; Reynaud et al., 2018). Some cross-sectional studies based on self-report data from adolescents find that sleep duration was not significantly associated with EF (Anderson et al., 2009) and at least one study has reported a negative association between sleep duration and EF (Lv et al., 2020). Some experimental studies also find that adolescents who are assigned to sleep restriction conditions do not perform worse in EF tasks than those in non-restriction conditions (Beebe et al., 2019; Suppiah et al., 2016; Voderholzer et al., 2011). The inconsistent findings regarding sleep and EF could be a result of the specific measures of sleep and EF that have been used across studies (Dewald et al., 2010; Reynaud et al., 2018). However, the variation in findings across these variable-focused investigations could also be due to unassessed but meaningful variation in person-specific sleep needs or reactivity to sleep changes (Banks & Dinges, 2007; Belenky et al., 2003; Van Dongen et al., 2003). In other words, the association between EF and sleep may vary from person to person, and an “average” effect for a sample may mask the person-specificity in the within-person relations between sleep and EF.

Between-person approaches cannot detect person-specificity in the relations between sleep and EF. When researchers draw conclusions from between-person analyses, it is implicitly assumed that the relation between EF and sleep is equivalent across individuals in the sample (e.g., Molenaar & Nesselroade, 2015; Rose, 2016). However, some youth might need more sleep than others, or some youth might have EF functioning that is more sensitive to changes in sleep compared to others. To the extent that these conjectures are true, group-based conclusions drawn from between-person differences in EF and sleep may not be appropriate to apply to any specific individual. Between-person analyses of the EF-sleep link do not illuminate relations when a specific youth sleeps better than usual, worse than usual, or in a manner consistent with the individual’s specific average sleep regimen (Molenaar, 2008; Molenaar & Nesselroade, 2015; Rose, 2016).

Some experimental studies did capture within-person fluctuations in EF and sleep by manipulating sleep quality in youth and found that children and adolescents tended to perform worse on EF tasks after sleep restriction (Carskadon et al., 2011; Fonseca & Genzel, 2020; Huang et al., 2016; Jiang et al., 2011; Lo et al., 2016; Randazzo et al., 1998). However, most studies report the “average” within-person effects, which still mask the person-specificity in the sleep-EF relations. In other words, we do not know from these studies whether the coaction between sleep and EF is the same across individuals. To optimize EF and effective learning for every child, “one-model-fit-all” strategies may not be impactful due to the heterogeneity in individual needs and reactivity. Thus, researchers should examine the person-specificity in the within-person relations between EF and sleep to build the foundation for effective person-specific interventions.

To our knowledge, only one study collected intensive repeated data on sleep and working memory among children across weeks and analyzed within-person relations between sleep and EF while accounting for person-specificity (i.e., random effect in multilevel modeling; Könen et al., 2015). The study found that, on average, if a child sleeps better than usual compared to themselves, they tended to perform better in working memory tasks, and the within-person associations varied across individuals. However, this study only included the working memory component of EF. More studies are needed to examine the person-specific within-person relation between sleep and more than one indicator of EF among school-aged children and adolescents. The present study was undertaken to provide such information.

## The Current Study

To generate the information about the relation between EF and prior night’s sleep within individuals, intensive repeated within-person measurements and ipsative analyses are needed (Bolger & Laurenceau, 2013; Lerner, 2018; Molenaar, 2008; Rose, 2016). To balance the person-specificity and generalizability of the findings, we apply a multilevel framework instead of a pure idiographic approach. We will apply a multilevel framework with intensive longitudinal data to separate the within-person variability and between-person variance. We will use the random effect of the relations between sleep and EF to indicate the person-specificity in the within-person relations. To compare participants with themselves, sleep at the within-person level will be centered with individual means to only link within-person fluctuations in sleep to EF performance. We sought to answer three questions:

(1) Is the prior night’s sleep associated with the subsequent day’s EF performance?

(2) Did the person-specific relation between prior night’s sleep and EF performance vary across individuals and differ from the group mean?

(3) Was the person-specific relation between sleep and EF moderated by individuals’ age and average sleep quality?

## Methods

### Participants and Procedure

Participants were recruited from elementary, middle, and high school classrooms across Massachusetts, Texas, and Washington, D. C. in the United States. Convenient sampling occurred as a multi-stage process in which the research team first contacted schools and classroom teachers. Once a teacher agreed to participate, all students in their classroom were offered the opportunity to participate. Both participants’ assent and parental consent were obtained before data collection. A total of 108 participants from Grades 4 to 12 were initially enrolled. Within-person variability in EF was assessed within an intensive longitudinal design, which is a design involving many repeated measurements within a short period of time (Bolger & Laurenceau, 2013).

Beginning early in 2020, participants used an online platform to complete measures between one and four times per week, primarily in the classroom during regular school instruction. In March 2020, in-school data collection was halted because of the COVID-19 pandemic restrictions. Accordingly, to have sufficient time points to enact person-specific analyses, we restricted our analytic sample to participants who completed at least 20 measurement occasions, which we regarded as a sufficient number of occasions for meaningful analyses and interpretation of within-person relations. Accordingly, the final analytic sample included 73 participants who provided data on at least 20 measurement occasions (*M* = 40.26, *SD* = 9.09). The data was collected across approximately one semester (*M* = 155.26 days, *SD* = 27.48). The ages of the participants ranged from 9.75 to 18.08 years old (*M* = 14.44, *SD* = 2.52). Most participants were boys (59.2%). Among the participants, 32.4% were Black/African American, 47.9% were Latinx, 14.1% were mixed race, and 5.6% were European American. In regard to maternal education level, 28.3% of the mothers had less than high school degrees, 26.7% had high school degrees or GEDs, 36.7% had some college, associate, or trade school degrees, and 8.3% had a 4-year college degree or higher.

### Measures

#### Executive functioning

Each time participants logged onto an online platform to participate, they were instructed to complete the Dimensional Change Card Sort (DCCS) task, the short Flanker task, and the Common Object Ordering (COO) task in a randomized order.

The short DCCS task primarily measures the cognitive flexibility component of EF (Yu et al., 2021; Zelazo et al., 2013). Participants were asked to select between two cards that matched a target object either by color or shape. Five color and five shape trials were randomized for each participant on each measurement occasion. There were two versions of DCCS, and game versions were randomly assigned to avoid boredom. Accuracy and reaction time were measured for each trial. Trials with reaction times shorter than 200 msec or longer than 3 *SD*s above the individual’s daily mean or 3000 msec were excluded due to inattention (Finch et al., 2019; Miyake et al., 2000; Sulik & Obradović, 2018; Zelazo et al., 2013). An accuracy score was computed as the percentage of correct trials multiplied by the number of total trials, ranging from 0 to 10. Both accuracy and reaction times were then used to compute the overall DCCS score due to a potential accuracy-reaction time trade-off (Zelazo et al., 2013). Median reaction time was first log-transformed and then algebraically rescaled to a range between 0 and 10. Participants with less than an 80% accuracy rate kept their accuracy score as the final DCCS score, and participants with 80% or higher accuracy rate were scored using the sum of accuracy and reaction time score as the final DCCS score (Zelazo et al., 2013). The range of the DCCS final score was 0 to 20.

The short, self-administered version of the Flanker task primarily measured the response inhibition component of EF (Huizinga et al., 2006; Yu et al., 2021). Participants were asked to identify the direction of the target object in the center of a row of objects. The task consisted of 12 trials: 6 congruent and 6 incongruent trials. In congruent trials, the center object was facing the same way as the rest of the objects, and, in incongruent trials, the center object faced the opposite way as the rest of the objects. In addition, the row of objects moved across the screen. In congruent trials, the motion was in the same direction as the target object, and, in incongruent trials, the motion was in the opposite direction as the target. There were two versions of the Flanker task, and game versions were randomly assigned to avoid boredom. Similar to the DCCS task, median reaction time and mean accuracy were computed for incongruent trials and congruent trials, respectively. Trials with reaction times shorter than 200 msec and trials with reaction times more than 3 daily *SD*s above the child’s daily mean or 3000 msec were excluded due to inattention. The accuracy score is based on the total of congruent and incongruent accurate trials (ranged from 0 to 12), and the median reaction time score was only based on the incongruent trials and log-transformed and then algebraically rescaled to range between 0 and 12. When the accuracy rate fell below 80%, the accuracy score was the final score. When the accuracy rate was 80% or above, the final score was the sum of accuracy and reaction time scores. The range of final inhibition scores was 0 to 24.

The COO tasks primarily measured the working memory component of EF (Yu et al., 2021). Participants were shown a series of pictures of common objects (e.g., banana, back-pack) and asked to re-order them in the order they were shown. Participants were asked to order 3 pictures in Trial 1, 5 pictures in Trial 2, 7 pictures in Trial 3, and 9 pictures in Trial 4. The participants were not asked to order the objects as fast as possible because the ordering process is likely to be impacted by the characteristics of the device being used. Therefore, only accuracy was assessed. Among all participants, individuals were able to order the objects correctly in the first trial. Due to increasing difficulty for each trial, different weights were given to the four trials. The working memory score was computed as 1 × correct objects in Trial 1 + 2 × correct objects in Trial 2 + 3 × correct objects in Trial 3 + 4 × correct objects in Trial 4. At the end of each trial, feedback on correctness was provided. The score ranged from 0 to 70.

#### Sleep

Sleep can be measured via multiple dimensions, and the most commonly used tools measures sleep quantity and quality (e.g., Buysse et al., 1989; Pilcher et al., 1997; Williams et al., 2013). In intensive longitudinal studies, it is important to limit the number of items to reduce participants’ response burden and therefore to help promote engagement and compliance (Heron et al., 2017; van Roekel et al., 2019). At the same time, the wording of the items need to reflect daily changes. Self-reported sleep quality was found to be most representative of the global sleep quality score (i.e., a comprehensive score of sleep quality, sleep latency, sleep duration, habitual sleep efficiency, sleep disturbances, sleep medication, and daytime dysfunction) in the Pittsburgh Sleep Quality Index (Buysse et al., 1989). Moreover, studies have found that sleep quality might be a better predictor of health and well-being than sleep quantity (Pilcher et al., 1997). Particularly, Könen et al. (2015) found that sleep quality was predictive of the next morning’s working memory performance.

Due to the extensive involvement required from the participants, we decided to implement a single item to measure subjective sleep quality. Previous scholars have successfully adapted a single item of subjective sleep quality to capture day-to-day fluctuations in perceived sleep quality (e.g., Tracy et al., 2020). In our study, we modified the item wording to be suitable for children and adolescents. Prior night’s sleep was measured via a single item “How well did you sleep last night?” The participants can choose from: 1 = *Terrible*, 2 = *Poorly*, 3 = *OK*, 4 = *Well*, and 5 = *Very Well*.

#### Covariates

Child age was collected from the consent form and included as the between-person level covariates. Time, defined as days passed since the first measurement occasion, was included as a within-person covariate to control for potential trends in EF performance.

### Analysis plan

Prior to answering the research questions, we first conducted preliminary analysis, including descriptive statistics, zero-order bivariate correlations, and intraclass correlations (*ICC*s) to examine the relations among study variables and the origin of variance. Before estimating the relations between sleep and EF, a 2-level confirmatory factor analysis (MCFA) was first used to create within-person (Level 1) and between-person (Level 2) common EF latent scores using Mplus 8.2 (Muthén & Muthén, 1998–2017). The exploratory analysis demonstrated that game versions of DCCS and Flanker tasks were associated with the performance, so game versions were included as covariates for the observed indicators of DCCS and Flanker performance, respectively, at Level 1. Moreover, the cumulative measurement occasion and cumulative measurement occasion square were included as covariates to account for a potential non-linear trend in the performance due to practice effects for all three EF components at Level 1. The within-level latent factors reflect the structures of within-person fluctuations in EF, and the between-person factor loadings describe between-person differences in personal average EF.

To evaluate the model fit of the MCFA, a maximum likelihood (ML) estimator was used at first. Model fit was evaluated based on comparative fit index (CFI), Tucker-Lewis index (TLI), root mean square error of approximation (RMSEA), and standardized root mean square residual (SRMR). An RMSEA < .08, a CFI > .90, and TLI > .90 indicate good model fit (Brown, 2006). However, ML does not produce standardized coefficients in multilevel modeling. To obtain standardized factor loading coefficients, the Bayesian estimator in Mplus 8.2 (Muthén, & Muthén, 1998–2017) was used. All models that used Bayesian analysis produce distributions of coefficients instead of a single parameter. For the purposes of easy interpretation, the median of the parameter distribution and the 95% credible interval *(CrI)* are reported in the results. If zero is not included in the 95% *CrI*, this finding can be interpreted as a 95% probability that the true estimate does not contain zero and is considered “statistically significant” in the conventional frequentist sense. Compared to ML estimation, Bayesian estimation has been shown to have a better ability to overcome convergence problems, lower estimation bias, and better flexibility in Multilevel Structural Equation Modeling^1^ (MSEM; Depaoli & Clifton, 2015; Muthén & Asparouhov, 2012; Stegmueller, 2013).

Two MSEM models were conducted to answer the three research questions. To answer the first two research questions, the first MSEM model was conducted in Mplus 8.2 (Muthén, & Muthén, 1998–2017). Mplus decomposes the sleep predictor into a within-level sleep (clustered-centered) and a between-level sleep (individual average sleep across all measurement occasions). The outcome was the daily latent EF score, and the main predictor was the sleep quality of the night prior to EF task performance. At the within-person level (Level 1), sleep quality was centered on individual mean sleep quality to reflect the within-person comparison. The cluster-centered within-level sleep can be interpreted as within-person fluctuation compared to their usual sleep quality. The exploratory analysis did not find a linear trend in EF or sleep; therefore, time was not included as a covariate at the within-person level. The equations are:

Level 1:

$$EF_{ij}=\beta_{0j}+\beta_{1j}(Sleep_{ij}-{\bar{Sleep}}_{j})+r_{ij}$$

Level 2:

$$\begin{array}{c} \beta_{0j}=\gamma_{00}+\gamma_{01}{\bar{Sleep}}_{j}+\gamma_{02}Age+\mu_{0j}\\ \beta_{1j}=\gamma_{10}+\mu_{1j} \end{array}$$

In the equations, *i* stands for any given day and *j* stands for any given individual. The *EF_ij_* is the individual latent factor score on a given day. The *Sleep_ij_* is the individual’s sleep on a given day, it is centered using the individual mean of sleep quality ${\bar{Sleep}}_{j}$. At the between level, *β_0j_* is the random intercept, which differed between individuals can be potentially explained by average sleep quality and age. *β_1j_* is the random slope of EF and sleep, and the error term *μ_1j_* represents the variance of the random slope. A significant error term (*μ_1j_*) implies that there is a significant variation in the relation between EF and sleep across individuals.

The third research question is whether age and average sleep can moderate the relation between prior night’s sleep quality and subsequent EF performance. To answer this question, a second MSEM model was conducted. Individual average sleep quality was added to the random slope equation at Level 2 and age was added as a covariate. To make the intercept of the random slope interpretable, sleep was first centered using the grand mean. The intercept can be interpreted as the mean slope when the predictor is grand-mean centered. The equations become:

Level 1:

$$EF_{ij}=\beta_{0j}+\beta_{1j}(Sleep_{ij}-{\bar{Sleep}}_{j})+r_{ij}$$

Level 2:

$$\begin{array}{l} \beta_{0j}=\gamma_{00}+\gamma_{01}{\bar{Sleep}}_{j}+\gamma_{02}Age+\mu_{0j}\\ \beta_{1j}=\gamma_{10}+\gamma_{11}{\bar{Sleep}}_{j}+\gamma_{12}Age+\mu_{1j} \end{array}$$

In this model, *γ_11_* and *γ_12_* are the moderation effect of individual average sleep on the random slope and the error term becomes the residual variance of the random slope, which is not explained by the cross-level moderator.

## Results

### Preliminary Analysis and 2-Level Confirmatory Factor Analysis

Zero-order descriptive and bivariate correlations were computed for all study variables (see Table 1). Three EF tasks were correlated with each other. ICCs of the three EFs ranged from .30 to .41, meaning that 30% to 41% of the EF task variance came from between-person differences, and the rest of the variance came from within-person variabilities. The MCFA has a low loading on the working memory score. The zero-order correlations between sleep and three EF tasks were weak for Flanker and close to zero for DCCS and Flanker tasks. Age was positively correlated with three EF tasks but negatively associated with sleep. This finding meant that, in general, older participants had better performances for the three tasks but reported lower levels of sleep quality.

**Table 1:** Descriptive Results and Zero-Order Correlation of Study Variables

		*M (SD)*	ICC	1	2	3	4	5	6
1	DCCS	12.57 (3.56)	.30						
2	Flanker	16.84 (3.06)	.37	.38**					
3	COO	41.37 (17.97)	.41	.26**	.23**				
4	Sleep	3.37 (1.15)	.36	-.05**	-.10**	-.01			
5	Age	14.55 (2.53)	-	.19**	.27**	.14**	-.10**		
6	DCCS Version	0.50 (0.50)	-	.04**	-0.01	.00	-.02	.02	
7	Flanker Version	0.50 (0.50)	-	-.00	-.08**	-.02	-.01	.01	.00

* *p* < .05,

** *p* < .01.

*Note*. DCCS = Dimensional Change Card Sort. COO = Common Object Ordering. ICC = Intra-class correlation.

**Figure 1:**
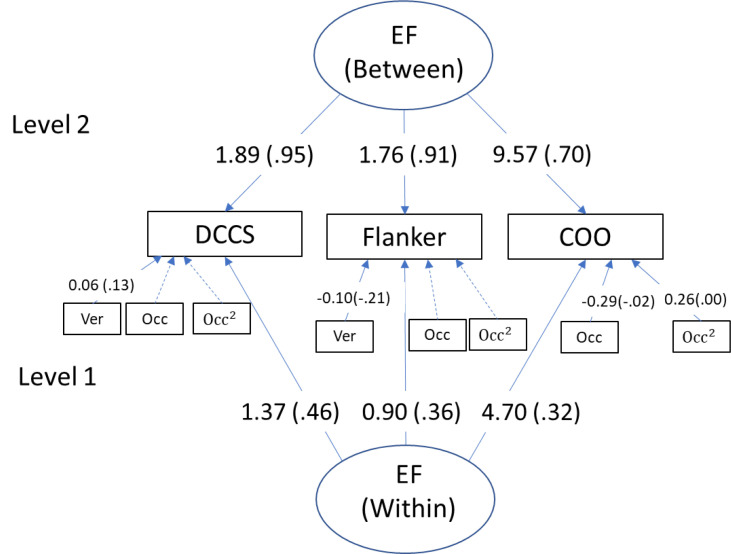
Unstandardized and standardized (in parentheses) coefficients using Bayesian estimation are shown. Only “significant” coefficients are shown. Ver = Game version, Occ = cumulative measurement occasions, Occ^2^ = square of cumulative measurement occasions. A separate MCFA model using ML yielded a similar results and good model fit indices: Chi-square (df)= 2.42(4), p = .66; CFI = 1.00; TLI = 1.06; RMSEA = .00; SRMR (within) = .01; SRMR (between) = .001.

Using ML estimation, the 2-level CFA model demonstrated good model fit (see Figure 1). The standardized factor loadings were then estimated using Bayesian estimation (reported in Figure 1). ML and Bayesian yielded similar unstandardized factor loadings. Three tasks had higher loadings at the between-person level than the within-person level. The between-person EF common factor loading supported a common EF factor based on between-person differences, which was consistent with existing evidence that EF sub-components are interrelated based on between-person differences (e.g., Garon et al., 2008; Miyake et al., 2000; Miyake & Friedman, 2012). At the within-person level, the lower factor loadings suggest that the tasks captured a lower level of reliable within-person variability compared to between-person differences.

### Random Intercept Random Slope MSEM Model of EF and Sleep

Building upon the 2-level CFA model, multilevel path models were then conducted. According to the first MSEM model (see Table 2), on average, prior night’s sleep quality was not significantly associated with EF performance on the next day. However, the variance of the sleep-EF coefficient was significant, implying heterogeneity in the relations between sleep and EF across all the individuals. The sleep-EF coefficients were assumed to be distributed normally. Building upon this assumption, coefficient one *SD* above the mean was 0.02 + 0.49 = 0.51, and coefficient one *SD* below the mean was 0.02 - 0.49 = 0.47. Such a significant random effect implies that the relations between sleep and EF were different for different individuals.

**Table 2:** Unstandardized coefficient in Multilevel SEM Model of Executive Functioning and Sleep

	*B*	*CrI*
Within		
Sleep (S1)	0.02	[-0.13, 0.17]
Variance S1	**0.24**	**[0.14, 0,41]**
Between		
Average sleep	-0.36	[-0.73, 0.002]
Age	**0.12**	**[0.07, 0.18]**
DIC	57614.296	
pD	309.582	

*Note. Cr*I = credible intervals. DIC = Deviance information criterion. pD = effective number of parameters. Bolded parameters can be interpreted as “significant.” DIC is a relative model fit index with smaller number indicating a better model fit. DIC balances the model fitness with a penalty for the number of parameters expended. pD is the number of parameters used in the penalty.

### Cross-Level Moderation of Average Sleep

In the second MSEM model, individuals’ age and average sleep quality were entered as a predictor for the random slope of within-person EF and sleep (see Table 3). Personal average sleep quality but not age moderated the within-person relations between EF and sleep. If a participant, on average, sleeps one SD above average sleep quality, the within-person sleep and EF coefficient would be -0.07 + (-0.26) × 1.15 = -0.37. If a child sleeps one SD below average sleep quality, the within-person sleep and EF coefficient would be -0.07 - (-0.26) × 1.15 = 0.23. In other words, if a participant usually sleeps better than the others, a better night of sleep compared to themselves was associated with worse EF performance. However, if a participant usually sleeps poorly, a better night of sleep was associated with improved EF performance. In other words, the relations between sleep and EF depend on the usual sleep quality one individual gets.

**Table 3:** Unstandardized coefficient in Multilevel SEM Model with Average Sleep as a Between-Person Moderator

	*B*	*CrI*
Within		
Sleep (S1)	-0.07	[-0.88, 0.76]
Residual variance of S1	**0.22**	**[0.13, 0.39]**
Between		
Average sleep	-0.34	[ -0.72, 0.04]
Age	**0.19**	**[0.12, 0.25]**
S1 × Average sleep	**-0.26**	**[-0.45, -0.01]**
S1 × Age	0.01	[-0.05, 0.06]
DIC	57611.647	
pD	309.478	

*Note. Cr*I = credible intervals. DIC = Deviance information criterion. pD = effective number of parameters. Bolded parameters can be interpreted as “significant”. DIC is a relative model fit index with smaller number indicating a better model fit. DIC balances the model fitness with a penalty for the number of parameters expended. pD is the number of parameters used in the penalty.

For a more straightforward interpretation of the results, we conducted a simplified complementary analysis to illustrate the findings. According to the multilevel CFA model, the DCCS tasks was the strongest indictor for EF. Therefore, the DCCS task was selected as the indictor for EF in the simplified analysis. The person-specific bivariate correlations between EF and sleep were computed using each person’s repeated measurements as a data set. The person-specific correlations showed a wide range from -0.67 to 0.51, with 0.03 as the mean and 0.21 as the SD. To echo the moderation effect, the bivariate between-person correlation between personal mean sleep quality and the person correlation coefficients was computed, *r* = -0.17, p = 0.17. Although not significant, all the coefficients were consistent with the trends of coefficients in the MSEM model, implying that higher average sleep quality was associated with smaller within-person correlation coefficients between sleep and EF.

## Discussion

EF describes a series of complex cognitive functions that support goal-directed behaviors and effective learning (Blair & Ursache, 2011; McClelland et al., 2014; Miyake & Friedman, 2012; Schmitt et al., 2017). Existing research on EF relies primarily on between-person approaches, emphasizing between-person differences in EF and related factors. However, EF may also vary within individuals, and the within-person variations might contribute to day-to-day learning experiences. To optimize learning on a daily basis, it is important to understand how EF change from day to day together with other factors in the developmental context (i.e., prior night’s sleep).

The current study employed an intensive longitudinal study design and multilevel framework to examine how participants’ prior night’s sleep contributed to daily fluctuations in EF (Bolger & Laurenceau, 2013; Lerner, 2018; Molenaar, 2008; Rose, 2016). We found that, on average, prior night’s sleep was not associated with daily EF within individuals. However, the within-person relation between sleep and EF varied across individuals. Average sleep but not age can partially explain the heterogeneity in the within-person relation between sleep and EF. For participants who usually sleep better than the others, a better night of sleep was associated with worse than usual EF performance, whereas for participants who usually sleep worse than the others, a better night of sleep was associated with better than usual EF performance.

### Moving from the Average Effect to Person-Specific Effects

Existing literature disproportionally emphasizes results that are generalizable across individuals (i.e., differential or nomothetic generalizations; Lerner, 2018). Such generalizations build on the assumption that the relations between sleep and EF are the same across individuals. This study highlighted that an average within-person association was not appliable to different individuals in the sample. Although the average within-person relation was not statistically significant, it would have been incorrect to draw the conclusion that sleep and EF were not covarying from day to day for everyone in the sample. Indeed, the heterogeneity in the within-person relations between sleep and EF implied that one model did not fit all participants. Such a finding might be a reason why there is inconsistency in findings of the relations between sleep and EF using between-person approaches or average within-person effects (e.g., Anderson et al., 2009; Lv et al., 2020). The heterogeneity in the sleep-EF coaction is consistent with ideas derived from the RDS metatheory. Each individual develops through dynamic and mutually influential relations between the individual and the multi-layered ecology of the individual; the person-specificity in their ecology contributes to the heterogeneity in the within-person sleep-EF effect (Bronfenbrenner & Morris, 2006; Molenaar, 2014; Overton, 2015; Witherington, 2015).

Adding between-person predictors helped explain the heterogeneity in the within-person relations between EF and sleep. For participants who normally have higher-than-others quality of sleep, a better-than-usual night of sleep is associated with worse-than-usual EF performance. In turn, for participants who normally have lower-than-others quality of sleep, a better-than-usual night of sleep is associated with better-than-usual EF performance.

One plausible explanation is that self-reported sleep quality partially measured total sleep time and that sleep quantity and quality might be positively correlated. Sleep quality is highly correlated with a global sleep quality score in the Pittsburgh Sleep Quality Index (Buysse et al., 1989), which includes measurements of sleep quantities. Moreover, sleep quantity and sleep quality showed similar predictive effects of well-being despite the different effect sizes (Pilcher et al., 1997). Thus, such a finding aligns with previous findings on the inverted U-shape of people’s need for sleep length to optimize cognitive functioning (e.g., Könen et al., 2015). In other words, too much or too little sleep are not ideal for optimal cognitive functioning. However, the criteria for “too much” can be person-specific (Rose, 2016; Rose et al., 2013). Thus, this study contributed to the current literature interrogating sleep-EF relations by using a multilevel approach and comparing within-person sleep fluctuations to the personal average, which is a personalized criterion for “too much” sleep.

Another possible explanation is that, for individuals who usually sleep well, a better-than-usual sleep quality might be linked to a very low level of stress and arousal. According to the bidirectional model of self-regulation, the performance of EF is a result of the integration of both cognitive and physiological processes (Blair & Raver, 2012b; Blair & Ursache, 2011; Obradović, 2016). EF is reduced at both very high and very low levels of physiological arousal but optimized at moderate levels of arousal (Blair & Ursache, 2011; Obradović, 2016). For individuals with high level of mean sleep quality, a better-than-usual sleep may reduce the physiological arousal to a very low level and reduce EF performance. Future research interrogating these possibilities should include measurement of physiological arousal to examine whether physiological arousal mediates the relation between sleep quality and EF.

The study contributes to the more general literature emphasizing the importance of including an idiographic component in all developmental research (Molenaar, 2004). Every human has attributes that are shared by all humans (nomothetic attributes), attributes shared with only some other attributes (group differential attributes), and attributes unique to the individuals (idiographic attributes) (Allport, 1937; Kluckhohn & Murray, 1948; Lerner, 2018; Molenaar & Nesselroade, 2015; Rose, 2016; von Eye, et al., 2015). That is, if the study of human development is to fully understand the holism of human development and to move towards using this knowledge to describe, explain, and optimize the life course of each person, the dynamic coaction of idiographic, differential, and nomothetic features of each person must be integratively studied (Cantor et al., 2021).

Accordingly, in this context, it is important to reiterate that this study’s within-person findings are not a replication of findings based on between-person differences in overall sleep quality and overall EF. Both sleep and EF were conceptualized as dynamic constructs that fluctuate within-personally, and the relations between sleep and EF represent the covariation on a day-to-day timescale. In other words, the present results extend the literature on EF and sleep in children and adolescents by linking within-person fluctuation in prior night’s sleep to EF performance in a naturalistic setting. Such a within-person finding is an additional piece of supportive evidence that within-person daily EF fluctuation is meaningfully associated with contextual and personal factors. However, at the between-person level, we did not find an association between EF and sleep, meaning that among this sample of participants, if an individual reported a higher level of quality in general compared to others, the person did not perform better in EF tasks compared to others. Such a finding suggests that comparing each individual to themself might be a more informative way to predict a specific individual’s development than comparing a specific individual to others (Cantor et al., 2021).

### Limitations

This study used an intensive longitudinal design, multiple sources of data, and behavioral EF measures. We view these features of our method as strengths of the research because they allowed us to appropriately address our primary research questions. However, despite these strengths, this study had several limitations. One of the major limitations was the use of a relatively small convenience sample. In addition, more evenly spaced and frequent occasions of measurement (i.e., identical timepoint divisions within and across participants and more time points for all participants) would have yielded a more accurate representation of each individual’s sleep quality and EF performance and allow the models to account for the temporal patterns of sleep and EF. In addition, sleep quality was measured using only one item to facilitate data collection, thus, limiting the content validity of the measure. Future studies should use measures or devices that accurately assess several components of sleep quality including the precise duration and a participant’s level of sleepiness throughout the day. We also did not include contextual factors. In order to fully capture the context, studies evaluating sleep and EF performance can potentially include measures that assess the child’s home environment, which was an omission of measurement that limited the present study.

Adding between-person predictors helped explain the heterogeneity in the within-person relations between EF and sleep. For participants who normally have higher-than-others quality of sleep, a better-than-usual night of sleep is associated with worse-than-usual EF performance. In turn, for participants who normally have lower-than-others quality of sleep, a better-than-usual night of sleep is associated with better-than-usual EF performance.

### Conclusions and implications

Person-specific relations between sleep and EF cannot be represented by an “average effect” or the results simply based on between-person differences. Between-person differences in personal average sleep quality can explain the heterogeneity in such within-person relations. Students whose sleep quality is below average are likely to benefit from a good night of sleep. On the contrary, for students whose sleep quality is above average, a better-than-usual night of sleep may hinder their EF performance. The findings highlight the person-specificity in both fluctuations in EF and relations between sleep and EF. The person-specificity implies that a “one-model-fit-all” program may fail to address the needs of each individual, resulting in ineffective intervention. Understanding the daily variation in sleep and EF performance allows for a more complete analysis of the whole student and constitutes knowledge that may facilitate interventions targeted at improving academic performance, as well as other components associated with EF performance and better sleep quality (e.g., mental wellness, behavioral adjustment, and physical health).

With the goal of optimizing effective learning for each student, within-person and person-specific evidence are more compelling than between-person correlational findings on sleep and EF (Bornnstein, 2019; Cantor et al., 2021). The findings suggest that improving sleep quality can be a target for intervention on daily EF for students who usually experience poor sleep quality. Intervention program developers, practitioners, and educators can shift from using group-based evidence to individual-specific evidence to inform practices in educational settings. In this way, decisions can be made based on each individual’s specific strengths, potentials, and needs instead of their deviation from averages on a single (or sparsely assessed) assessment.

## Footnotes

1 We decided not to use a nonlinear dynamic systems approach to try to account for the temporal pattern of sleep and EF because we were primarily interested in the concurrent relation between sleep and EF. Moreover, the relatively small number of measurement occasions and the fact that the time intervals between measurement occasions were not even created problems for computing temporal dependency.

## Action editor

Lars-Gunnar Lundh served as action editor for this article.
